# Hungry for biomarkers of aging

**DOI:** 10.1111/acel.14158

**Published:** 2024-03-27

**Authors:** Nathan K. LeBrasseur

**Affiliations:** ^1^ Robert and Arlene Kogod Center on Aging Mayo Clinic Rochester Minnesota USA; ^2^ Paul F. Glenn Center for Biology of Aging Research at Mayo Clinic Rochester Minnesota USA; ^3^ Department of Physical Medicine and Rehabilitation Mayo Clinic Rochester Minnesota USA

Greater insights into biological mechanisms (*hallmarks*) of aging, their associations with aging‐related diseases and disabilities, and early evidence that they can be modified by intervention have generated intense interest in testing the *geroscience hypothesis*; that is, interventions targeting hallmarks of aging will delay or even prevent age‐related diseases and disabilities. This innovative and potentially disruptive approach to optimize human health has revealed the need, potential, and challenges for operationalizing biological markers, or *biomarkers*, of aging.

Sensitive and specific methods continue to progress for quantifying the loss of proteostasis, mitochondrial dysfunction, epigenetic alterations, oxidative damage, cellular senescence, and other hallmarks of aging (Lopez‐Otin et al., 2023) at tissue and single cell levels, particularly in preclinical models. Their utility in humans, however, can be limited by the accessibility of tissues most vulnerable to age‐related conditions, for example, heart, brain, lung, and kidney. This challenge has driven the National Institute on Aging (https://predictivebiomarkers.org/), a recently formed Biomarkers of Aging Consortium (https://www.agingconsortium.org/), and other entities to develop feasible, valid, and, in the context of interventions targeting specific hallmarks, modifiable biomarkers of aging (Moqri et al., 2023, 2024; Rutledge et al., 2022). As an example, we exploited the literature and the secretome of several human cell types induced to senescence in vitro to develop a candidate panel of senescence biomarkers, comprised of cytokines, chemokines, matrix remodeling proteins, growth factors, and other proteins, that could be reliably measured in the blood of humans (Schafer et al., 2020). We have observed expected changes in their circulating abundance in conditions associated with increased senescent cell burden, including advanced chronological age, frailty, disability, and chronic disease (Aversa, Atkinson, et al., 2023; Fielding et al., 2023; Schafer et al., 2020). Most recently, we studied biospecimens and clinical data from the Comprehensive Assessment of Long‐term Effects of Reducing Intake of Energy (CALERIE™) study and demonstrated that participants randomized to 2 years of calorie restriction had reduced concentrations of senescence biomarkers at Year 1 and Year 2 compared to those randomized to an ad libitum diet (Aversa, White, et al., 2023). In the thoughtful categorization of biomarkers as *response*, *predictive*, *or surrogate*, proposed by Cummings and Kritchevsky, the change in the circulating abundance of senescence‐related proteins in response to calorie restriction would support their use as *response biomarkers* (Cummings & Kritchevsky, 2022).

Response biomarkers of aging hallmarks are critical for the advancement of pharmacological interventions targeting mechanisms of aging. In part, such biomarkers can provide confirmation in early‐phase clinical trials of target engagement (e.g., reduction in the burden or secretory phenotype of senescent cells or reprogramming of the epigenome to a more youthful state) and guide optimal dose and dosing regimens. If the field is to be so fortunate to have biomarkers of aging hallmarks and an arsenal of pharmacological interventions to target distinct, albeit interrelated, mechanisms of aging, these biomarkers may also foster personalized medicine and the selection of the right intervention, for the right person, at the right time. However, several hurdles on the track to translation persist. For example, whether circulating biomarkers of senescence or other hallmarks reflect an average state of aging across all organs or are more strongly influenced by the biological age of a particular organ is unclear. Similarly, whether accessible response biomarkers are informative of change in a target organ (e.g., the lungs in the context of a pulmonary disease) in response to an intervention remains to be demonstrated. A recent study showing that sets of circulating proteins can be linked to specific organs highlights the potential to overcome such barriers and for our field to develop accessible biomarkers of aging that are reflective of a given tissue (Oh et al., 2023).

Another key question is whether changes in the abundance or activity of a hallmark of aging in response to an intervention will also be predictive of changes in longer term clinical outcomes of interest to regulatory agencies, that is, how one feels, functions, or survives. This is a reasonable potential application of biomarkers of aging but, at this early stage, largely remains unanswered. In more than 1900 relatively healthy older adults with either zero or one condition at baseline, our panel of senescence biomarkers was able to predict mortality better than the combination of age, sex, race, and the presence of a chronic condition (St Sauver et al., 2023). Moreover, in CALERIE™ study participants, we recently reported that changes in the circulating abundance of senescence biomarkers were highly associated with changes in the homeostatic model of insulin resistance and a calculation of insulin sensitivity (Aversa, White, et al., 2023). This would suggest that circulating senescence‐related proteins may also serve as *predictive biomarkers* and perhaps *surrogate biomarkers*, which respond to intervention *and* predict future health outcomes. A classic example of a surrogate biomarker is blood pressure. However, calorie restriction, like exercise, undoubtedly mediates improvements in metabolic health through multiple biological mechanisms, limiting causal attribution (i.e., elimination of senescent cells or suppression of their secretome is responsible for the clinical benefits of calorie restriction). Additional research is needed to determine whether changes in biomarkers of aging hallmarks in response to interventions that more specifically target senescent cells, genomic instability, deregulated nutrient‐sensing, or stem cell exhaustion, etc., can be predictive of recognized clinical outcomes such as metabolic, musculoskeletal, cardiovascular, immunological, and cognitive function, in line with the geroscience hypothesis.

Finally, it should be noted that biomarkers of aging hallmarks may offer utility for clinical decision making, even in the absence of interventions targeting those hallmarks. Individuals of advanced biological age presumably have heightened vulnerability to age‐related diseases and disabilities and poor outcomes in response to medical and surgical interventions (e.g., drug toxicity, complications, increased length of hospital stay, and rehospitalization). Indeed, biomarkers of senescence, mitochondrial dysfunction, DNA methylation, somatic mutations, and other hallmarks have shown potential for predicting risk for adverse events in older adults (Fielding et al., 2023; Jaiswal & Ebert, 2019; Mahapatra et al., 2023; McCrory et al., 2021). It is plausible for biomarkers of aging to be used in combination with existing risk calculators, which are now heavily influenced by *chronological* age, to inform clinical decisions, such as electing more conservative medical versus more aggressive surgical management of a condition, prescribing prehabilitation prior to an intervention, or proactively initiating comprehensive transitional care plans to prevent rehospitalization following discharge from the hospital.

Given the potential and promise, it is exciting to observe and contribute to the current multidisciplinary and collaborative efforts within our field to develop and test predictive, response, and surrogate biomarkers of aging. The expectation is that biomarkers of aging will help advance the geroscience hypothesis from a compelling premise to clinical practice.

## CONFLICT OF INTEREST STATEMENT

NKL and Mayo Clinic have intellectual property related to this work licensed to a commercial entity. This research has been reviewed by the Mayo Clinic Conflict of Interest Review Board and is being conducted in compliance with Mayo Clinic's Conflict of Interest policies.
